# EEG Radiotelemetry in Small Laboratory Rodents: A Powerful State-of-the Art Approach in Neuropsychiatric, Neurodegenerative, and Epilepsy Research

**DOI:** 10.1155/2016/8213878

**Published:** 2015-12-24

**Authors:** Andreas Lundt, Carola Wormuth, Magdalena Elisabeth Siwek, Ralf Müller, Dan Ehninger, Christina Henseler, Karl Broich, Anna Papazoglou, Marco Weiergräber

**Affiliations:** ^1^Department of Neuropsychopharmacology, Federal Institute for Drugs and Medical Devices, 53175 Bonn, Germany; ^2^Institute of Physiology, University Medical Center Mainz, 55128 Mainz, Germany; ^3^Department of Psychiatry and Psychotherapy, Faculty of Medicine, University of Cologne, 50924 Cologne, Germany; ^4^German Center for Neurodegenerative Diseases, 53175 Bonn, Germany

## Abstract

EEG radiotelemetry plays an important role in the neurological characterization of transgenic mouse models of neuropsychiatric and neurodegenerative diseases as well as epilepsies providing valuable insights into underlying pathophysiological mechanisms and thereby facilitating the development of new translational approaches. We elaborate on the major advantages of nonrestraining EEG radiotelemetry in contrast to restraining procedures such as tethered systems or jacket systems containing recorders. Whereas a main disadvantage of the latter is their unphysiological, restraining character, telemetric EEG recording overcomes these disadvantages. It allows precise and highly sensitive measurement under various physiological and pathophysiological conditions. Here we present a detailed description of a straightforward successful, quick, and efficient technique for intraperitoneal as well as subcutaneous pouch implantation of a standard radiofrequency transmitter in mice and rats. We further present computerized 3D-stereotaxic placement of both epidural and deep intracerebral electrodes. Preoperative preparation of mice and rats, suitable anaesthesia, and postoperative treatment and pain management are described in detail. A special focus is on fields of application, technical and experimental pitfalls, and technical connections of commercially available radiotelemetry systems with other electrophysiological setups.

## 1. Introduction

Within the last decades radiotelemetry became a widely used and highly recognized methodological* in vivo* approach for measuring a variety of behavioral and physiological parameters in conscious, unrestrained animals of various sizes, for instance, in the context of electroencephalogram (EEG), electrocardiogram (ECG), electromyogram (EMG), blood pressure, body core temperature, or activity measurements [1–7]. Virtually any species can be analyzed using radiotelemetry from laboratory rodents such as mice and rats to cats, dogs, pigs, and primates [3, 8]. Even fish, reptiles, and amphibians are subject to radiotelemetric investigation [9]. In the past, it has proven to be valuable in the characterisation of various transgenic animal models of human diseases, such as epilepsies, sleep disorders, and neurodegenerative and neuropsychiatric disorders [7, 10–12]. A number of methods collecting physiological data including biopotentials from mice and rats have been described. A special focus had been given on physical restraint methods, tethered systems, worn in jacket recorder systems, or nonimplanted radiotransmitters [13, 14]. Currently, a number of systems are commercially available for radiotelemetric implantation.

In general, telemetry recordings from conscious animals are thought to be superior to those from restrained or anesthetized animals, since they represent the normal physiological and behavioral state and are more predictive of the results that would be achieved in humans [1, 3]. Restraining methods can induce stress artefacts and severely influence basic physiological parameters, such as heart rate, blood pressure, body core temperature, and food intake [3]. A classical restraining approach in EEG recording is the use of tethered systems [13, 14] in which electrodes are connected to a miniature socket anchored to the skull and exposed for attachment of a cable. Although tethered systems allow relatively free movement of the animal, one of its major disadvantages is the fact that it is still semirestraining and that there is an increased risk of infection at the electrode implantation site.

Although wireless radiotelemetry technology for monitoring larger laboratory animals has existed for some time, it has only recently become affordable, reliable, and relatively easy-to-use [10, 15, 16], even for mice and rats. Smaller transmitters are now commercially available (e.g., TA10ETA-F20, ETA-F10; Data Sciences International, DSI, USA), which can be implanted in mice greater than 20 g (~10 weeks), so that animal size is no longer an absolute limitation. Indeed, transmitter size continuously decreases and companies develop systems with even more enhanced features. Implantable transmitter systems are capable of minimizing most disadvantages related to potential recording-associated stress by restraining systems. Mice are able to show a complete repertoire of physiological behavior including resting, locomotor activity (exploration), and sleep (REM, slow-wave sleep) [17, 18]. Furthermore, telemetry leads to a strong reduction of animal use [3]. Currently, there is an intense discussion on how to limit the number of experimental animals in science and to reduce their suffering. Clearly, animal experimentation and animal models of human and animal diseases are essential for our understanding of the pathophysiology and subsequent progress in therapy. Furthermore, animal experiments are a paramount in drug research and development and substantially contribute to preclinical/toxicological studies in drug licensing. No alternatives are yet available to animal experimentation to understand the complex interdependence of different neuronal subsystems which would be otherwise impossible to be illuminated. At the same time, the 3R, that is, replacement, reduction, and refinement strategy in the EU and the USA, strongly encourages research into complementary and alternative methods. Radiotelemetry is an example of a successful 3R strategy as it can reduce the number of experimental animals and their suffering compared to other techniques [3, 19, 20].

This is the first contiguous, detailed description of a successful, quick, and simple surgical procedure for s.c. pouch implantation of a radiofrequency transmitter in mice and rats and subsequent lead placement in both epidural and deep intracerebral positions. We discuss numerous commercial and self-assembled approaches in radiotelemetry and the pros and cons of different implantation techniques. In addition, we provide valuable information on equipment, fields of application, for example, spontaneous EEG recordings, circadian influence on central rhythmicity, sleep studies, that is, spontaneous sleep, pharmacologically induced sleep, sleep deprivation, and seizure analysis, and the combination of radiotelemetry with other techniques such as pump systems or auditory brainstem-evoked potential recordings. Furthermore, examples of high-quality electrocorticograms (ECoGs) as well as deep intracerebral recordings from mice and rats are displayed and special attention is paid to detecting, analyzing, and avoiding EEG artefacts. We also comment on frequent experimental pitfalls in radiotelemetry and strategies for reduction of trauma and optimization of pain management during postoperative recovery. Finally, we discuss approaches of EEG analysis.

## 2. Materials and Methods

### 2.1. Selection of Experimental Animals: Species and Strains

Quantitatively, most radiotelemetric studies are carried out in rodents, that is, mice and rats. Transgenic mouse models that fulfil the requirements of homology, isomorphism, and predictability related to a specific human disease entity are a primary subject of radiotelemetric methodology, particularly in cardiovascular and neurological research. Besides basic science, radiotelemetry has also become the method of choice in drug research and development including preclinical toxicological and mutagenicity studies. Radiotelemetry has been applied in a vast majority of mammals including cats, dogs, pigs, and primates, but also in other animal taxa such as fish, amphibians, or reptiles [7, 9, 21–23]. In this paper the scope of discussion will be the application of EEG radiotelemetry to small rodents.

It should be noted that the miscellaneous mouse and rat strains available can severely differ in basic physiological and pathophysiological characteristics that have to be considered or evaluated prior to performing subsequent electrophysiological experiments [24–26]. Mouse strains, for example, can exhibit dramatic discrepancies in their responding to applicable dosages of specific anesthetics [27, 28] necessitating pilot experiments to evaluate the most effective and secure anesthetic approach. In addition, it is known that mouse strains can display clear differences in sleep architecture and seizure susceptibility [29, 30], two fields that radiotelemetry is often applied to. Another nonnegligible issue is gender: the oestrous cycle can strongly affect central rhythmicity, its circadian dependency, and sleep as well as seizure activity [31–33]. In general, it seems recommendable to perform gender-specific analyses, or—if financial and experimental capacity is limited—to restrict to male mice as they are lacking confounding effects of the estrous cycle. If mixed gender study groups are unavoidable, both genders should be balanced. It should also be tested whether a gender-specific (re)analysis (*post hoc* analysis) of recorded data is possible. As electrophysiological* in vivo* studies using mice and rats are highly susceptible to a variety of confounders/disruptive factors, controllable parameters such as age and body weight should display small interindividual variability within a given study group. The same holds true for the housing conditions (see below).

### 2.2. Animal Housing and Pretreatment of Mice and Rats

Within an animal facility, mice and rats are normally housed in filter-top cages or even better in individually ventilated cages. We first transfer mice from the animal facility to ventilated cabinets that are placed in special lab rooms exclusively dedicated to house implanted animals and their subsequent recording. For acclimatization after ground transportation the animals are placed there for one week [34]. We use ventilated cabinets, such as Model 9AV125P (Tecniplast, Germany) and UniProtect cabinet (Bioscape, Germany). However, other cabinet models, for example, from Scanbur (Denmark), Alesco (Germany), and so forth, are also commercially available and enable temperature maintenance of 21 ± 2°C, 50–60% relative humidity, and a conventional 12 h light/dark cycle. In general, ventilated cabinets strongly support animal welfare but also help to provide stable environmental conditions. They offer a wide range of configurations and complimentary equipment and can be run with positive and negative pressure. Most importantly, ventilated cabinets reduce the time of exposure to microorganisms, dust, allergens, and other contaminants for animals as well as for personnel. As long as the ventilated cabinet is closed, such exposure is eliminated as all supply and exhaust air is cleaned through the highly efficient filters. As a result, the risk of infection of the animals inside the cabinet when the doors are opened is reduced. This risk can further be eliminated by transferring the cages to a laminar flow cabin for handling procedures. Doors of ventilated cabinets are transparent made of polycarbonate with magnetic closure and can be covered with red transparency film so that the circadian rhythm remains undisturbed from external light. Most importantly, ventilated cabinets provide excellent sound isolation. This is of central relevance as noise is a severe confounding element in almost any experimental setting using transgenic models [35–38]. Thus, standardized housing conditions are mandatory for actually all scientific questions, particularly for studies where circadian rhythmicity plays a major role, such as analysis of sleep architecture [39, 40].

Mice 12 weeks of age are used for most experimental settings; this, of course, depends on the scientific questions to be addressed. Prior to implantation (see below) mice are housed in groups of 3-4 in clear Macrolon cages type II (26.7 cm × 20.7 cm × 14.0 cm, area 410 cm^2^) with ad libitum access to drinking water and standard food pellets. Clear Macrolon cages type III (42.5 cm × 26.6 cm × 18.5 cm, area 800 cm^2^) are used for rats. The cage bottom bedding consists of wood shavings. It should be noted that even the cage bottom structure, namely, wire-bottom cages or bottom bedding can eventually affect heart rate, body weight, and locomotor activity due to stress reaction [41]. Animals should not be separated and placed individually at this stage as isolation could also cause stress influencing experimental results. However, following radiotransmitter implantation, animals should be housed separately as the animals tend to manipulate wound stiches/sutures or metal clips (see below). Clearly, open housing conditions do not seem to be appropriate for most scientific questions due to environmental disturbances of mice and rats based on, for example, running lab equipment, air-conditioning, or working lab staff. Open housing conditions are judged inappropriate for sleep studies, as they can result in a sleep deprivation setting rather than recording of physiological spontaneous sleep.

Whenever possible, mice and rats should be housed in separate rooms and separate ventilated cabinets. There should also be mouse and rat specific equipment so that neither mice nor rats can sense the presence of each other as this poses stress to the animals.

Finally, we would like to comment on the evolving issue of environmental enrichment and its relevance for radiotelemetry [42]. Environmental enrichment exposes laboratory animals to novelty and complexity through alterations in the physical and social environment, which can lead to enhanced sensory, cognitive, and physical stimulation. It should be noted that enriched housing strategies are a highly recommended practice by governing bodies regulating animal welfare. This is mainly due to the fact that enrichment causes benefits on rodents' well-being based on the more naturalistic environment [43]. In females, the impact of maternal enrichment on both offspring and maternal behavior and physiology also has to be considered. Studies revealed that the environment has significant impact: for example, smaller testing chambers and a sensory attenuation cubicle around the chamber reduced spontaneous physical activity in mice [44]. One major limitation of current experimental approaches is that most studies are conducted on unstimulated, sedentary animals that have unlimited access to food in the home cage. This can lead to metabolic and physiological compromise. Interestingly, environmental enrichment which enhances cognitive activity, sensory stimulation, and physical exercise has been shown to induce severe effects on central nervous system (CNS) activity and behavior in both wild-type and transgenic rodent models, relative to standard-housed littermate controls [45, 46]. In addition, environmental enrichment has beneficial effects outside the CNS, such as a reduction in excess body fat. It has been widely recommended that positive study results, for example, therapeutic interventions, which are found to show promise in standard-housed preclinical animal models should be subsequently tested also under conditions of enhanced environmental enrichment. This strategy helps to identify therapeutics which continue to show efficacy in housing contexts of superior environmental construct validity [45]. Fascinatingly, it has been demonstrated that exposure to environmental enrichment significantly improves learning and memory in aged mice and reduces the abundance of 5-hydroxymethylcytosine, a gene-environment interaction mediated at the epigenetic level [47, 48]. In general, it must be considered that recordings under enriched conditions might be less standardized and exhibit increased data variability.

In many cases the administration of preoperative medication is recommended to minimize unnecessary anxiety and stress. Preoperative stress and pain can be relieved, for example, by benzodiazepines such as diazepam or midazolam, which serve as minor tranquilizers. Benzodiazepines might be coapplied with injectable analgesics (see below).

All animal experimentation has to be performed according to the guidelines of the local Council on Animal Care. In most cases, researchers further have to certify that all animal experimentation was carried out in accordance with superior legislation, for example, the European Communities Council Directive of 24 November 1986 (86/609/EEC) or individual regional or national legislation. Specific effort has to be made to minimize the number of animals used and their suffering. In this regard, radiotelemetry plays an important role in the 3R, that is, replacement, reduction, and refinement strategy.

### 2.3. Radiotelemetry System

Several telemetry systems, for example, DSI, TSE systems (USA), Indus Instruments (USA), and Millar Inc. (USA), are commercially available and slightly differ in their functional and technical specifications. The system that is finally chosen by the user should depend on lab specific requirements and current and future research goals and experiments. One should also consider whether the telemetry setup is compatible with other electrophysiological systems so that both might be connected or extended (see connection of DSI System and the auditory recording system from Tucker Davis Technologies (TDT, USA) below).

Amazingly, a literature search revealed a total of 29 publications claiming the development of a self-made radiotelemetry device of substantial novelty that best meets the requirements of the users [49–77].

The most relevant advantage of self-made system as brain-machine interfaces pertains to their low costs. However, it has to be considered that one has to invest time and effort in developing self-made systems including complex analysis software later on. In most cases, a commercially available system is more expensive but might be more straightforward and is definitely time-saving.

**Figure 1:**
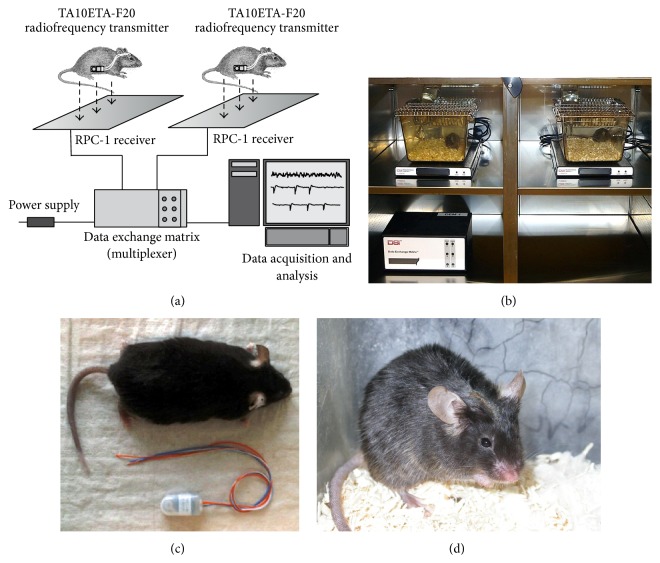
Standard EEG radiotelemetry system and radiofrequency transmitters. Besides self-made systems, a number of commercially available systems are on the market. The basic setup of such system is depicted in (a). The system consists of a radiofrequency transmitter, the receiver plate, a data exchange matrix serving as a multiplexer, and the data acquisition, processing, and analysing core unit. For frequency analysis, seizure detection and sleep analysis specific software modules are offered. Multiple types of transmitters are available depending on which species is supposed to be investigated and depended on the scientific question. (b) Implanted mice, receiver plates, and a multiplexer placed inside a ventilated cabinet for standardized recording conditions. (c) An adult C57Bl/6J mouse and a 2-channel radiofrequency transmitter (TL11F20-EET; DSI). (d) Dorsal view of the skull 4 weeks after electrode implantation and fixation using glass ionomer cement. Figures 1(a) and 1(d) reprinted from [85], with permission from Elsevier.

The telemetry system used in our lab is obtained from DSI. As a standard telemetry implant we use the one-channel PhysioTel transmitter TA10ETA-F20 (technical specification: 3.9 g, 1.9 cc, input voltage range ±2.5 mV; channel bandwidth (B) 1–200 Hz, nominal sampling rate (f) 1000 Hz (f = 5B), temperature operating range 34–41°C, warranted battery life 4 months, on-off mechanism magnetically actuated, DSI, Figure 1) capable of measuring biopotentials, that is, EEG, ECG, EMG, and physical activity and temperature. In addition, we also implant the two-channel transmitter TL11 M2-F20EET (technical specifications: 3.9 g, 1.9 cc, input voltage range ±1.25 mV, channel bandwidth (B) 1–50 Hz, nominal sampling rate (f) 250 Hz (f = 5B), temperature operating range 34.0 to 41.0°C, warranted battery life 1.5 month, on-off mechanism magnetically actuated). These two extra-small implants can be used in mice, hamsters, gerbils, and rats. It should be noted that a number of transmitter types are available that differ in the number of recording channels, their transmitter bandwidth and nominal sampling rate, physical dimensions, battery life span, and in situ battery exchangeability. Finally, the size of the chosen transmitter determines the minimum animal weight. For the TA10ETA-F20 and TL11 M2-F20-EET the minimum animal weight for an s.c. implantation is about 20 g; for i.p. implantation it is considerably higher (DSI). The reasons are discussed in detail below. Thus, specific care has to be taken in choosing the correct transmitter type for a specific scientific question to be addressed. There are multiple examples in the literature of transmitter type applications where transmitters were used out of the range of their technical specifications, for example, high-frequency gamma pseudo-recording by a transmitter that was not able to record this activity. At the worst this can result in recording of nonexisting data, that is, ghost data.

The signal entities are transmitted to a receiver, for example, RPC-1 (DSI), which picks up the telemetered data from the implant and forwards these data to a Data Exchange Matrix (DSI), the latter serving as a multiplexer.

### 2.4. Time Required for Implantation Procedure

In the following a detailed description of radiofrequency transmitter implantation and electrode positioning will be provided. The total duration for the implantation and electrode placement clearly depends on the number of EEG deflections and whether recordings are to be performed from the cortex or deep intracerebral brain structures. Well-trained scientists will manage to do surgery within 30 to 45 min. It turned feasible for beginners to practice implantation using euthanized mice or rats in order to speed up the implantation procedure and to get used to computerized stereotaxic implantation. A long-lasting implantation procedure, particularly long-lasting narcosis poses specific stress to the animal and leads to subsequent impairment of recovery with potential loss of the animal.

### 2.5. Surgical Instrumentation: General Aspects

Small rodents are predisposed to hypothermia due to their high ratio of body surface (mouse, 10.5 × (weight in g)^2/3^; rats, 10.5 × (weight in g)^2/3^) to body volume [78]. Thus, supplemental warmth has to be given during and after surgery using recirculating warm water blankets, electrical warming plates, heat lamps, forced warm air units, or pocket warmers to maintain body core temperature. The latter should be 36.5–38.0°C (98.6–100.4°F). In addition, to avoid corneal desiccation, eyes are to be covered with petroleum-based artificial tear ointment or dexpanthenol (Bepanthen, Bayer, Germany) during the whole implantation period and early recovery until the blinking reflex is totally restored. Further supplies include disinfectant, 2% glutaraldehyde solution (Sigma, Germany), 0.3%/3% hydrogen peroxide solution (Sigma), sterile drapes, tapes, sterile scalpel blades with handle, sterile gloves, sterile pads, sterile 0.9% saline (NaCl) or lactated Ringer's solution (prewarmed for injection), sterile cotton tip applicators, and nonabsorbable surgical suture material (5-0/6-0) for lead ligation. The following instruments, for example, from FST (Germany), turned out to be helpful, that is, tissue forceps 1 × 2 teeth (12 cm length), standard pattern forceps (12 cm and 14.5 cm length), lexar baby scissors (10 cm), Tungsten carbide iris scissors (11.5 cm), Halsey micro needle holder (15.5 cm), Iris scissors extra thin (10.5 cm), Olsen-Hegar Needle Holder-extra delicate, Graefe forceps-curved, serrated, and Graefe forceps-curved, with teeth and bulldog clamps. Surgical instruments can be autoclaved for sterilization or be placed in disinfectants. An elegant and fast way is using a heat-based surgical instrument sterilizer with glass beats (FSN, Germany). A binocular surgical magnification microscope and a cold light source, for example, KL2500 LCD (Schott, Germany), for intense illumination via flexible or self-supporting, movable light guides should be accessible. The experimenter should wear a clean laboratory coat, a facemask, a head cover, and sterile gloves. Clearly, optimal supplies and instruments vary from lab to lab and must meet lab-specific and institutional requirements.

### 2.6. Anesthesia

Rats and particularly mice present an anesthetic challenge because of their small size and varied responses to anesthetic drugs within strains and between genders [27, 28, 79]. Even animals of the same age can severely vary in body weight, fat distribution, and fat proportion, thus posing specific requirements to the dosage of injectable and volatile anesthetics due to differences in the distribution volume [80, 81]. This paragraph provides basic information for injectable anesthesia and inhalant anesthesia for both mice and rats. A common and well-established anesthetic injection regime consists of a combination of esketaminhydrochloride (Ketanest, Parke-Davis/Pfizer, Germany, 100 mg/kg) and xylazine hydrochloride (Rompun 2% Bayer, Germany, 10 mg/kg). Whereas ketamine as a predominant glutamate receptor antagonist severely dampens brain activity causing loss of consciousness [82, 83], xylazine serves as a potent muscle relaxant [79]. Although this dosage is usually sufficient for the implantation duration it turned out that different mouse and rat strains and in particular different transgenic mouse lines can exhibit totally different susceptibility towards ketamine/xylazine injection. Some (transgenic) mouse lines display an extremely narrow therapeutic anesthetic window, probably related to primary or secondary effects of transgenesis leading to either insufficient narcosis or overdosage and death, the latter often based on xylazine mediated lethal respiratory depression or cardiac arrest [83]. Other injectable anesthetics in rodents include pentobarbital, ketamine/xylazine/acepromazine, ketamine/medetomidine, or tribromoethanol. According to our experience, volatile anesthetics can be helpful in strains and lines that display increased and critical sensitivity to injectable anesthetics. Inhalation narcosis, for example, using isoflurane, sevoflurane, or halothane, can be controlled easily, particularly with regard to initiation, maintenance, and termination, that is, emergence/arousal of/from anesthesia [80, 81, 84]. The fast and almost immediate respiratory elimination of isoflurane (respiratory rate: mouse 150–220 breaths/min; rat 70–115 breaths/min; minute ventilation: mouse 24 mL, rat 200 mL [78]) guarantees fast recovery. The delivery of volatile anesthetics to rodents is usually done using a silicone facemask since in small size rodents endotracheal intubation is more difficult to perform than in larger species. The advantage of endotracheal intubation is that the animal can be artificially ventilated in the event of cardiac or respiratory arrest. However, it should be noted that attempts to intubate rodents, even by experienced personnel, often traumatize the oropharynx, larynx, trachea, and at times the oesophagus. In general, endotracheal intubation and artificial respiration are not required for mouse and rat anesthesia.

Using volatile anesthetics has a number of advantages over injectable anesthetics, for example, minimal animal handling, large margin of safety, ease of anesthetic control, low cost of the anesthetic drug, and use of no controlled drugs. Inhalation anesthesia has thus become the standard method of general anesthesia for mice and rats in biomedical research. Drawbacks of this approach are mainly restricted to the high initial costs of equipment including isoflurane vaporizer, supply gas (carbogen) and its regulator, a flowmeter, an induction chamber, tubing, face masks, and an isoflurane absorber (scavenging method). We use Isoflurane Baxter (100%V/V) in a Matrix TM VIP 3000 Calibrated Vaporizer and a scavenger system from Harvard apparatus (USA). For initiation of isoflurane anesthesia an incubation chamber is used.

### 2.7. Types of Transmitters and Technical Specifications

There are various providers that offer a huge variety of transmitter types, not only for biopotential recordings such as EEG, ECG, and EMG but also for pressure measurements (blood pressure and respiratory pressure), and other applications. Most transmitter types also provide information on body temperature (normal mouse body core temperature is 36.5–38.0°C and normal rat body core temperature is 37.0–38.0°C) and activity. With regard to body temperature one should be aware that only i.p. implantation is suitable for accurate measurement of body core temperature. Subcutaneous pouch implantation of the radiofrequency transmitter (see below) results in slightly decreased temperature values. Nevertheless, it provides valuable information particularly during the postsurgical recovery period. As the lifespan of a typical radiofrequency transmitter is limited it is recommended to make notes about the individual recording times so that one is aware when the battery is going to cease. It should also be considered that many radiofrequency transmitters are turned on via a magnetic switch; thus, even if they are not used the storage time is limited. Expiration dates should be checked carefully. As outlined in Section 2.3 the technical specifications of each transmitter type also have to be considered carefully and must fit the experimental and analytical tasks. Special attention has to be paid to the transmitter bandwidth (B) and nominal sampling rate (f).

### 2.8. Transmitter Implantation

Following proper anesthesia it is recommendable to remove body hair from the scalp using a shaver (Aesculap, Eickemeyer, Germany). The shaved areas can be scrubbed with Mercurochrome (Merbromin, Germany) or 70% ethanol as disinfectant. It should be noted that transgenic mice in particular might not tolerate disinfectants well and might exhibit skin irritation or inflammation of variable degree. Depilation cream should not be applied due to skin irritation and possible wound healing impairments later on.

Incisions sites can also be cleaned with disinfectant betaisodona standard solution (Mundipharma, Germany). The animal will be placed in a sterile field on a warming surgery table to avoid hypothermia of the animal during the surgical procedure. For good fixation the limbs of the animal can be carefully taped to the table. Usage of an operation microscope might be helpful during the whole procedure.

Using surgical scissors a 2 cm midline incision of the scalp from the forehead to the neck is made. Starting from the nuchal incision side a s.c. pouch is created along the lateral flank of the animal by blunt dissection. Once the pouch is established, the transmitter is placed subcutaneously inside the pocket with the sensing leads oriented cranially. One concern when implanting the radiofrequency transmitter in a s.c. pouch on the back is a potential reduction in signal strength (e.g., monitored by the Dataquest A.R.T. software, DSI) due to the increased distance of the transmitter from the receiver plate. Therefore, the transmitter is implanted subcutaneously at the flank close to the ventral abdominal region and fixed at the skin using the transmitter's suture tab and a single stich to avoid postoperative movement of the transmitter. In this position, a reduction in signal strength is not observed compared to the i.p. implantation procedure. It should be noted that after s.c. placement the telemetered temperature values do not represent the real body core temperature. Though temperature values are below body core values, the recording allows for monitoring the temporal development of s.c. temperature. Undoubtedly, the s.c. pouch implantation of the radiofrequency transmitter is the most recommendable one.

However, it is also possible to perform an i.p. radiofrequency transmitter implantation. The intraperitoneal implantation starts with a 1.5–2 cm midline abdominal incision, that is, laparotomy. The abdominal skin and wall are opened and both incision edges are held aside using a 5 mm wound retractors together with elastomers, fixators, and a base frame (FST, Germany). Next, the transmitter is placed into the peritoneal cavity on top of the gastrointestinal tract. Special care must be taken not to impinge the bladder or diaphragm. Anatomical forceps are to be preferred to surgical forceps, as they produce less damage to the tissue, particularly when manipulating the bowel. In rare cases, local bleeding from the abdominal wound edges can occur which can easily be staunched using a thermocauter (Heiland, Germany). Note that bleeding from the scalp incision can also be easily stopped by thermocautery. Next, both leads are tunneled through the abdominal wall at the cranial part of the incision using a 14-Gauge needle. A trocar together with a plastic sleeve can be tunnelled subcutaneously along the lateral thoracic wall to the intended position at the neck, where a second skin incision was made before. The trocar can be withdrawn from the sleeve and the leads are tunnelled through it. Alternatively, a thin trocar with a short silicon tubing attached at its tip can be used to tunnel the leads subcutaneously to the neck. In this case, the sensing electrodes can be tied mechanically to the silicon tubing or by attaching them with glue. This method is much faster to perform and causes less damage to the s.c. tissue, which is important for rapid postoperative recovery. Leads are secured on the underlying muscle layer using a nonabsorbable interrupted stay suture (Ethilon^*∗*^II, 4-0, M-2, Ethicon, Germany). If not tacked, the intrinsic elasticity of the electrodes will make implantation of the electrodes more difficult later. The transmitter is fixed in the peritoneal cavity using its suture tab to avoid intra-abdominal movement and possible impingement. Finally, the abdominal wall is closed using nonabsorbable suture material (Ethilon II, 4-0, M-2, Ethicon, Germany). However, the ventral skin incision is closed using wound clips (Michel, 7.5 × 1.75 mm, Heiland, Germany), as mice tend to manipulate and bite sutures. Wound clips normally drop off later during the wound healing process (see [85]).

Generally, the intra-abdominal implantation is much more difficult to perform and takes longer time even for an experienced surgeon than the s.c. approach and in addition poses specific risks of pressure necrosis of the gut or even blockage of the stomach or bladder. Recently, [86] analyzed the effects of i.p. radiotelemetry instrumentation in mice revealing that i.p. implantation can size-dependently depress activity, reduce voluntary running, and lead to inflammation of the diaphragm, large intestine, and duodenum. Concomitantly, it delayed postsurgical recovery and disrupted normal growth. As the wellbeing of the animal is significantly affected by i.p. implantation, the data quality might also be affected [86]. For i.p. implantation it has also been reported that increased intra-abdominal pressure can reduce the venous blood flow back to the heart and increase vagal activity. This can lead to reduced heart rate in i.p. versus s.c. pouch implanted mice [85]. In 2004, Leon et al. 2004 analyzed the effects of body- (B-) to-transmitter (T) (B/T) size ratio on growth and circadian rhythmicity. A large B/T ratio was associated with a minimization of the adverse effects of i.p. implantation [87]. It becomes obvious that in small experimental animals such as mice implanted transmitters can alter activity, behavior, circadian rhythmicity, and sleep in a strain dependent manner.

### 2.9. Stereotaxic Device

**Figure 2:**
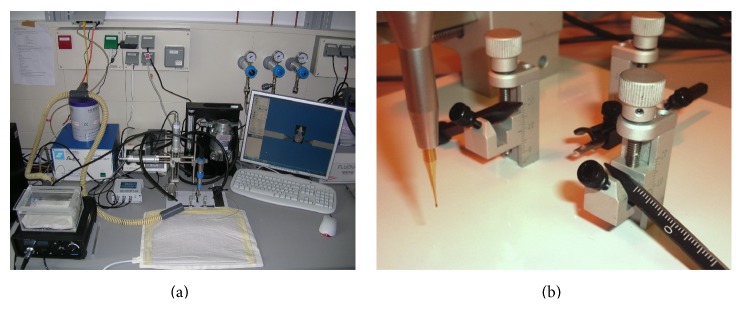
Anesthesia and stereotaxic setup for mice and rats. (a) Gas anesthesia system using isoflurane. A precision high-speed dental drill is mounted on a 3D stereotaxic device for mice and rats, respectively. Supplemental warmth is given using a heating pad. (b) Close-up of drill, stereotaxic ear bars, and nose clamp.

We use two special, fully equipped stereotaxic setups for mice and rats (Figure 2) from Stoelting (USA) and Neurostar (Germany). The stereotaxic device includes the stereotaxic frame with ear bars and tooth clamp size-adapted for mice and rats, respectively. In addition, the stereotaxic frame also includes a gas anesthetic mask (Stoelting, USA) with connections to the isoflurane evaporator and the isoflurane scavenger module. Gas anesthetic masks can also be self-made. The system serves as a computerized 3D stereotaxic setup with a specific software (StereoDrive; Neurostar, Germany) including a user interface for navigation and 3D atlas, allowing axial, coronal, and sagittal views. The setup enables reproducible and precise electrode placement according to stereotaxic coordinates. As in our case the high-speed precision drill is mounted on the vertical arm of the stereotaxic frame. In some cases, researchers mount pencils or pens on the vertical arm leaving a tiny mark at the coordinates of choice on top of the skull. Afterwards the animal is removed from the stereotaxic frame and holes are drilled manually. Clearly, stereotaxic drilling is much more precise and mandatory in small rodents like mice. Manual handling of a high-speed drill might be possible in rats but is not precise and dangerous. One should be aware that mice and rats severely differ in the neurocranial bone thickness. The thickness of the murine cranial bones depends strongly on the localisation (*os frontale*: midline section: 320–390 *μ*m, lateral section: 300–430 *μ*m;* os parietale*: midline section: 210–250 *μ*m, lateral section: 200–210 *μ*m;* os occipitale*: midline section: 600–730 *μ*m, lateral section: 380–420 *μ*m). Damage of the inner ear can be avoided by covering ear bars with cotton balls. These precautions allow for a tight fixation of the head within the stereotaxic frame. For some experimental purposes, like screening for audiogenic seizures or seizure susceptibility, this is of especially high importance. Ideally, holes should be drilled pressure-free at maximum velocity. This avoids a tonic applanation of the skull, which may result in a sudden breakthrough of the drill head and potential damage mainly in the cortical field. For craniotomy, we use the Zeppelin neurosurgical high-speed precisionmotor drill system ZMM-100e from Adeor Medical Technologies (Germany).

### 2.10. Stereotaxic Surface Electrode Implantation: ECoG

**Figure 3:**
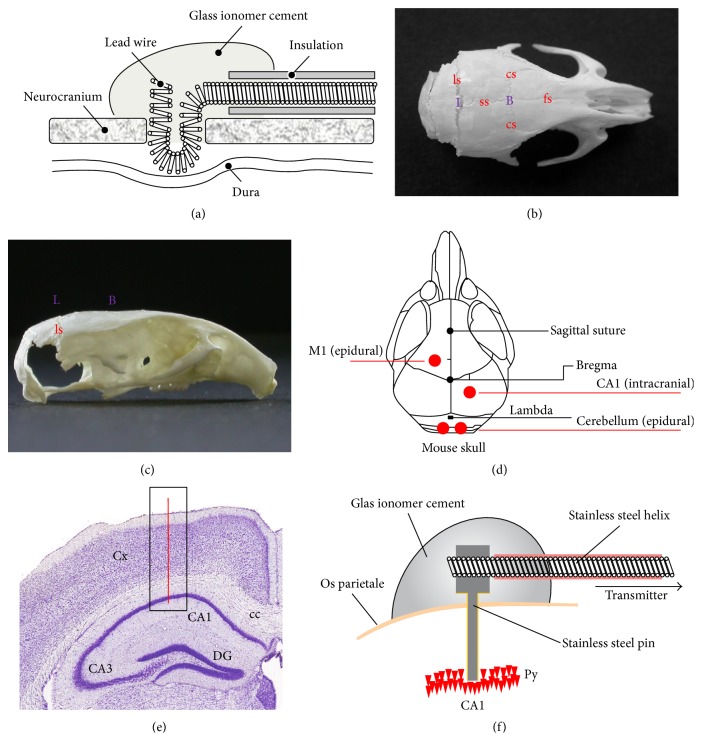
Stereotaxic surface and deep electrode implantation. (a) Scheme of an epidural electrode placement in mice and rats. (b) Anatomic structures and landmarks of the murine skull. Apical view of a C57Bl/6J mouse skull which has been prepared in 0.3% H_2_O_2_. Note cranial bones (os frontale (of), os parietale (op), and os occipitale (oo)) and sutures (sutura frontalis (sf), sutura sagittalis (ss), sutura coronaria (sc), and sutura lambdoidea (sl)) which determine the major anatomic landmarks bregma (B) and lambda (L). (c) Lateral view of a C57Bl/6J mouse skull. (d) One epidural, differential electrode is placed on the motor cortex (M1), and an additional intrahippocampal differential electrode is placed in the CA1 region of the hippocampus. Both pseudo-reference electrodes are localized on the cerebellum. (e) Coronal section (scheme) illustrating the localization of the deep, intracranial electrode for recording the electrohippocampogram. (f) Close-up of the deep EEG electrode, the sensing lead of the radiofrequency transmitter, and their arrangement on top of the murine skull.  Figure 3(b) reprinted from [85], with permission from Elsevier.

A midline skin incision, 10 mm on the head and 5–10 mm down the neck, is made and the s.c. tissue is bluntly separated. The periosteum is cleaned using cotton tips without damaging the temporal and occipital muscles. The superficial thin tissue layer of the skull is pretreated with 0.3% H_2_O_2_ for the mouse skull and 3% for the rat skull. This pretreatment removes any tissue from the skull and assures strong contact of the glass ionomer cement (Kent Dental, Kent Express Ltd., UK) which is used later on. It also accentuates the sutures as well as bregma and lambda landmarks of the skull, which are essential for precise stereotaxic lead placement (Figures 3(a)–3(d)). It should be noted that this procedure can produce severe oxidative damage to the surrounding tissue. As pointed out before, the mouse neurocranium is extremely thin and interspersed with osseous canaliculi. Thus, care must be taken not to incubate H_2_O_2_ for too long as this can lead to penetration of H_2_O_2_ through the skull and oxidative damage of the underlying cortex. Following the bleaching of the skull, burred holes are drilled at the coordinates of choice using a high-speed drill with typical drill head diameter of 0.3–0.5 mm. The diameter of the holes might be smaller depending on the electrode diameter. As a general rule, the smaller the diameter, the less damage is produced. Haemorrhage reflecting intraosseous bleeding from the cut edge of the skull can be easily staunched with a cotton tampon and is self-limiting in general. Fatal bleeding was never observed as positioning of the electrodes should differ from the localisation of dural sinuses. However, once a dural sinus has been hit, the survival rate of the animals is dramatically reduced. It seems recommendable to sacrifice these animals.

Holes are drilled at the coordinates of choice. The surface electrodes are shortly bend at the tip and placed directly on the dura mater for epidural lead placement, for example, in a bipolar deflection or (pseudo) unipolar deflection. As an example, a surface motor cortex M1/M2 electrode is positioned at: cranial 1 mm, lateral 1.5 mm (left hemisphere). An epidural reference electrode can be placed on the cerebellar cortex, for example, bregma −6 mm, lateral of bregma 1 mm (left hemisphere) or bregma −6 mm, and lateral of bregma 1 mm (right hemisphere), respectively (Figure 3(d)), as the cerebellum serves electroencephalographically as a silent region. It is often claimed in the literature that this procedure can be carried out without damage of the dura. However, given the small dimensions it seems questionable whether this really holds true. Next, electrodes were fixed with glass ionomer cement (Kent Dental, Kent Express Ltd., UK), which is extremely hard and gives strong adhesion to the underlying neurocranium. After the cement has dried (5 min), the scalp is closed using over-and-over sutures with nonabsorbable 6-0 suture material (Ethilon polyamid, Ethicon, Germany) (Figure 1(d)). Following implantation and fixation of the tip of the sensing lead using glass ionomer cement, no severe inflammatory reactions either of the cerebrum, the cerebellum, the meninges, or skin have been observed. In rare cases, increased pressure caused by the underlying cement resulted in skin necrosis during the observation period of 4–6 weeks; thus, an optimum amount of cement should be used to guarantee best electrode fixation and lowest s.c. pressure. It should be noted that it is also possible to place cortical screws and attach the sensing leads of the radiofrequency transmitter afterwards. This requires the use of connectors being a potential source of signal noise.

### 2.11. Stereotaxic Deep Intracerebral EEG Electrode Implantation

The scalp and skull of the animal are pretreated as described above. For deep electrode implantation holes are drilled at the stereotaxic coordinates of choice. Special attention has to be paid to the electrode material characteristics and its connection to the transmitters sensing leads. We use stainless steel or tungsten electrodes which are parylene coated (14.5 cm original length, 250 *μ*m diameter, 50–100 KOhm tip impedance, epoxylite insulation metal microelectrodes, FHC Inc., USA). First, the electrodes have to be shortened to the required length. An additional part of the electrode is required to connect it to the stainless steel helix of the transmitter lead. These two sections will be bent to a 90° angle in between. This square angle is important so that the transmitter lead can divert horizontally from the skull towards the transmitter. It reduces pressure on the skin and helps to fixate the electrode and the lead to the skull. Next, the stainless steel helix of the transmitter lead is exposed by removing a short section of the silicone isolation at the tip of the transmitter lead using a sterile scalpel blade. By rewiring the lead of the transmitter to the deep brain electrode, a suitable connection of both components is ensured. In general, soldering is not recommended as this can result in severe noise in the recordings. Once the solid electrode is connected to the transmitter lead, the electrode can be attached to the vertical arm of the stereotaxic device to lower it to the target brain region. When the target position is reached the electrodes are fixed using glass ionomer cement (Kent Express, UK). The CA1 region is one example of a deep electrode target. In order to reach this hippocampal region of interest, CA1 holes are drilled and positioned at the following coordinates referring to the bregma: caudal 2 mm, lateral 1.5 mm (right hemisphere), and dorsoventral (depth) 2 mm (see Figures 3(d)–3(f)). An epidural reference electrode can be placed on the cerebellar cortex, for example, bregma −6 mm, lateral of bregma 1 mm (left or right hemisphere) (Figure 3(d)). Once the electrodes are placed and attached to the sensing leads, the scalp is closed using over and over sutures (Ethilon, 6-0).

Note that stereotaxic coordinates are subject to changes during postnatal brain and skull development. In addition, there is intraindividual variability in the anatomical localisation of the major craniometric landmarks bregma and lambda [88, 89]. This holds true for both mice and rats.

### 2.12. Types of EEG Deflection

In general, one can differentiate between monopolar and bipolar deflections. In the monopolar deflection the differential electrode is placed at the localisation of choice whereas the reference electrode can be placed on a silent region of the brain, for example, the cerebellum. Special care has to be taken when using bipolar deflections as these can result in extinction of recorded EEG activity when activity of high bilateral synchronicity is present. This holds true for spontaneous absence like activity with characteristic spike-wave discharges or spike-wave discharge activity that has been induced pharmacologically, for example, via GBL (GHB) [90, 91].

### 2.13. Postoperative Care and Pain Management

After implantation, animals are placed back into their home cage and within the first 3-4 days after surgery body core temperature is maintained using a thermal lamp, warming plate, or heating pad. As the ratio of body surface to body weight is high in small rodents (36 cm^2^/20 g in mice), external temperature support was not removed until mice were able to maintain physiological body core temperature themselves. This can be monitored easily using the transmitter temperature sensing function if technically available.

For pain management several injectable analgesics are available: narcotic opioids, opioid agonists/antagonists, *α*_2_ agonists, local anesthesia, and nonsteroidal anti-inflammatory drugs (NSAID), that is, buprenorphine, butorphanol, tramadol, flunixin, ketoprofen, metamizole, meloxicam, carprofen, acetaminophen, and lidocaine [80, 81]. For example, metamizole-sodium (Novaminsulfon-ratiopharm 1, ratiopharm, Germany), which is used for postoperative pain management at 100 mg/kg body weight for four days after surgery can be administered intraperitoneally in Ringer solution or 0.9% NaCl. One of the disadvantages of metamizole is that it has to be administered repetitively due to its short half-life. Thus, we normally use 50 mg/mL carprofen (Rimadyl, Pfizer, Germany) diluted in Ringer solution or 0.9% NaCl for postoperative analgesia.

Postoperatively, animals are fed moistened pellets in order to facilitate food uptake. Food consumption (~15 g/100 g/d; ~5 g/24 h) and water consumption (~15 mL/100 g/d; ~5 mL/24 h) should be monitored carefully. No matter which transmitter implantation site is chosen, the maximum loss in body weight is around days 4-5 after surgery [85]. In addition, the animals should be closely monitored for the return of their normal postures and behaviors.

Systemic administration of antibiotics such as enrofloxacin or trimethoprim-sulphonamides [80, 81] is often recommended but is not performed in our studies and no inflammatory signs of meningitis or encephalitis at the sites of implantations were detected after surgery or at postmortem examination of any mouse implanted. In general, animals recover quickly and are given 10 to 14 additional days to fully recover before starting EEG recordings for further analysis. This recovery period is based on the observation that 10 days after surgery no difference in physiological parameters between transmitter implanted, nonimplanted, and sham-operated animals could be detected [3]. It should be noted however that special experimental settings, for example, behavioral tasks, demand for longer recovery periods, especially when performing i.p. implantation [92]. It is highly recommendable to use an animal implantation and documentation sheet to document animal recovery.

Postoperative recovery after i.p. placement of the transmitter can be evaluated by monitoring postsurgical development of body weight. A maximum reduction in body weight was observed around days 4-5 after surgery followed by a slight, but steady increase of weight during a 10–14-day recovery period after which it remained constant. The time pattern of recovery is in line with the postoperative development of body weight following i.p. implantation for ECG recording.

### 2.14. Validation of EEG Electrode Placement

To verify the correct electrode placement targeting, for example, the CA1 region, brains are extirpated postmortem and fixed in 4% paraformaldehyde. Afterwards, brains are cut to 60 *μ*m slices using a Vibroslice Tissue Cutter EMS 5000-MZ (Campden Instruments Limited, UK) and hematoxylin or Nissl stained for visualization of the branch canal.

### 2.15. Reuse of Radiofrequency Transmitters

Commercially available transmitters are sterilized in advance. In most cases transmitters can be reused and implanted repetitively. Thus, it is strongly recommended not to cut the sensing leads to the minimum length right from the beginning. Instead, the flexible, full-length leads should be placed subcutaneously as they do not impair the animal. Following euthanization, transmitters are explanted. Special care has to be taken not to impinge or damage the sensing lead insolation. Using a biodetergent, for example, Neodisher (Medizym, Germany), for removing tissue debris (24 h at RT) and 2% glutaraldehyde (Roth, Germany) for resterilisation (at least 2 h at RT), the transmitters are prepared for reimplantation. For removing all traces of chemical sterilant, especially glutaraldehyde, the transmitters are temporarily stored overnight in sterile saline or water.

### 2.16. Stability of EEG Recordings

The lifespan of the radiofrequency transmitter battery restricts the time of continuous recordings. In some systems, the battery and the transmitting unit of the radiofrequency transmitter are connected via a socket meaning that once the battery is discharged it can be replaced by a new one following a small cutaneous longitudinal incision. In another system an external recharge via induction is possible. According to our experience, high-quality EEGs can be reliably recorded for up to 8 weeks at least. Ossification from the drilled holes has the capability to lift up the electrodes with time resulting in EMG and ECG contamination, thus limiting optimum recording duration to a few months.

### 2.17. Signal-to-Noise Ratio

EEG recording systems making use of a tethering system require special connectors to attach the sensing leads to the implanted wire and might thereby introduce significant noise to the system. Radiotelemetry systems using screw electrodes [18] or isolated stainless steel wires for implantation [10] might therefore exhibit similar issues. In our recordings following implantation the signal-to-noise ratio ranged from 100 nV to 1 *μ*V. As the EEG signal is weak, it is highly susceptible towards ECG and EMG contamination, both of which can evolve into a severe problem (Figure 8). This often occurs if the sensing lead is not properly secured or isolated from extracranial tissues by glass ionomer cement or if the silicone insulation of the sensing leads is damaged during the implantation procedure or later on due to manipulation by the animal. ECG contamination can be easily identified due to its very regular pattern (R-spikes, i.e., heart rate in mice 300–650 beats/min (5–10.8 Hz) and 250–370 beats/min (4–6.2 Hz) in rats. Note that breathing can also result in slow EEG wave artifacts. Typical respiratory rates in mice are 150–220 breaths/min (2.5–3.7 Hz) and 70–115 breaths/min (1.2–1.9 Hz) in rats.

### 2.18. EEG Data Acquisition and Fields of Application

**Figure 4:**
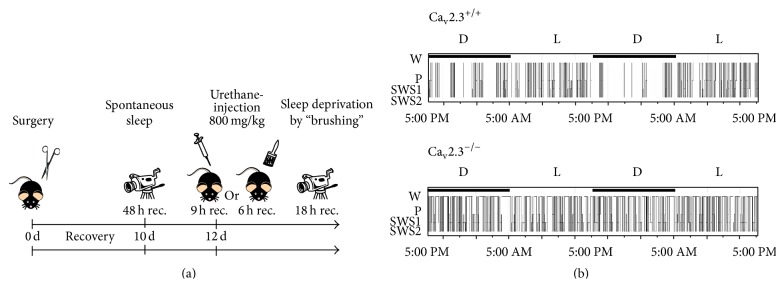
Application of EEG radiotelemetry, analysis of sleep architecture. (a) Typical time scale illustrating long-term recording of spontaneous sleep, pharmacologically induced sleep using urethane, and sleep deprivation. (b) Sleep analysis is an important tool in characterization of circadian aspects of central rhythmicity in control and transgenic mice. This figure depicts representative hypnograms (W, wake state; P, paradoxical sleep; SWS1, slow-wave sleep 1; SWS2, slow-wave sleep 2; L, light cycle; D, dark cycle) from a control and a transgenic mouse lacking the Ca_v_2.3 R-type voltage-gated Ca^2+^ channel [94].  Figure 4(b), reprinted from [94], with permission from Associated Professional Sleep Societies, LLC.

Ten days after radiotransmitter implantation, simultaneous video-EEG recordings from the motor cortex (M1) and the hippocampal CA1 region are performed for long-term recordings, for example, 48 h in all animals from both study groups using Dataquest ART 32 bit operating system with Dataquest A.R.T. 4.31 Gold version, Data Exchange Matrix and a Video Network Camera system (Axis P1343 Video Camera including day/night capability, Sweden). Different types of recordings can be performed depending on the scientific question to be addressed. Common fields of application comprise initial spontaneous EEG recording. It should be noted that there is a circadian dependency of central rhythmicity. Thus, recordings should either be done at the same day- (or night-) time or be preceded by an entire circadian analysis of rhythmicity. Another important field of application is sleep analysis. Sleep analysis does not only include spontaneous (at least 24 h) recordings, but also include pharmacologically induced sleep, for example, via urethane and sleep deprivation studies (Figure 4). Furthermore, a predominant field of application is seizure analysis and recording of evoked potentials.

### 2.19. Combining EEG Radiotelemetry with Other Scientific Approaches

#### 2.19.1. Radiotelemetric Recordings under Chronic Drug Application

In many experimental settings it is necessary to apply drugs for longer time periods. This holds true especially for antiepileptic drugs and neuropsychiatric drugs. One potential approach would be daily drug application via s.c. or i.p. injection. However, this turns out to be impracticable. Instead one can make use of, for example, s.c. implantation of osmotic minipumps (e.g., Alzet^(R)^, USA). Osmotic pumps are prepared 24 h in advance under sterile conditions and stored in saline solution (0.9%) at 37°C until implantation. Animals are anesthetized using isoflurane (2-3%) and a small incision is made on the back of the animal. For rats, small s.c. pouches are made on both sides of the cut edge adding up to the size of the pump plus additional space for movability.

For mice it is recommendable to make a small cutaneous incision between the shoulders so that the animal cannot reach the stiches with its teeth. Afterwards, a small s.c. pouch is made towards the back of the animal including additional space for movability.

The pouch is flushed with sterile saline solution, the osmotic pump placed inside, and the cut sewn with a few stitches. If the animal has the chance to manipulate the stiches with its teeth, wound clips should be used instead. As for radiofrequency transmitter implantation, Rimadyl (5 mg/kg) is administered for pain relief. Besides osmotic pumps, mechanical pumps are also available, for example, SMP-200 from iPrecio (USA). Pumps can deliver drugs for one up to six months.

#### 2.19.2. Auditory Research in EEG Radiotelemetry

**Figure 5:**
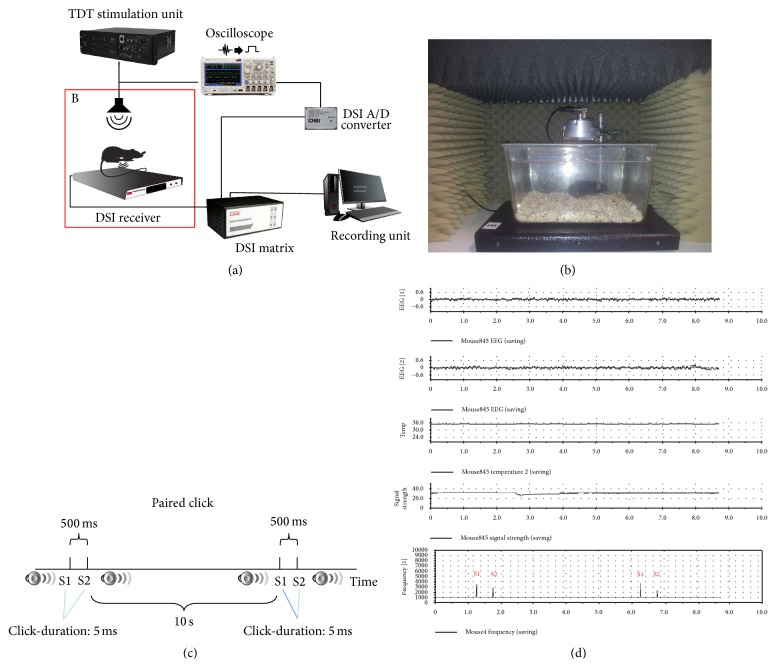
Auditory research using EEG radiotelemetry. Implantable EEG radiotelemetry can be used to record auditory evoked potentials. As depicted in (a), the TDT stimulation unit is connected to a loudspeaker and via an oscilloscope to an A/D converter. The data exchange matrix does not only receive EEG data via the receiver plate but also receive a trigger signal from the A/D converter that is integrated into the EEG recording. (b) Auditory setup with loudspeaker inside a sound isolation chamber. The radiofrequency transmitter implanted mouse is placed on the receiver plate inside the chamber. (c) Scheme of the paired click paradigm protocol. As illustrated in (d), EEG channels are synchronized with the double click trigger signal (S1, S2) enabling complex averaging processes for subsequent studies of auditory evoked potentials.

In order to record auditory evoked potentials (AEPs) from specific brain areas, one can use an auditory stimulation unit, for example, a RZ6 Multi I/O Processor system of TDT and the BioSigRZ software (TDT). The stimulus presentation and equipment control are coordinated using the RZ6 system and BioSigRZ software (TDT) (Figure 5).

The stimulus is presented via a loudspeaker (MF1 Multi-Function Speaker, TDT) placed centrally above the cage. A second output signal is transferred to an oscilloscope (DPO 3012, Tektronix, Inc., USA) and displayed. The oscilloscope is used as a trigger unit to exhibit a rectangular signal each time an acoustic stimulus is presented. The trigger signal is transferred to an A/D converter (C11V; DSI) and forwarded to the DSI Data Exchange Matrix via a Local Area Network type cable. The data exchange matrix multiplexes data signals from the active receiver (RPC-1, DSI) and the A/D converter combines both signals and sends the signal stream to the data acquisition system (Dataquest A.R.T. system DSI). All auditory recordings should be performed in a sound-attenuated recording chamber placed inside a Faraday cage for electrical isolation.

Illustrating all potential experimental combinations with EEG radiotelemetry would be beyond the scope of this presentation. However, we want to point out that various other valuable settings are possible, including, for example, the combination with behavior and cognition test regimes or intracerebroventricular cannulation and application settings [16].

## 3. EEG Radiotelemetry: Practical Application

### 3.1. Baseline Recordings from Mice and Rats

It is generally recommendable to perform long-term baseline recordings following a 10–14-day recovery period. Baseline recordings, for example, allow for detection of potential implantation-related artifacts, the description of spontaneous time-frequency characteristics of surface or deep EEG recordings [93], the circadian influence on rhythmicity [94], and spontaneous or induced seizure activity [95–97].

### 3.2. Seizure Activity following Acute Administration of Proconvulsive/Psychoenergetic Drugs

**Figure 6:**
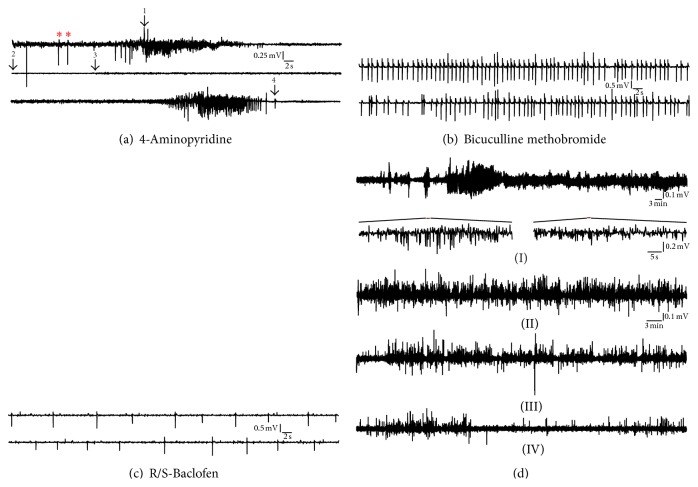
Pharmacological induction of epileptic discharges. (a) Surface EEG recording displaying ictal discharges after i.p. administration of 4-aminopyridine (4 AP, 10 mg/kg). Sporadic spikes (T) evolve into a transitory episode of continuous spiking (1), resulting in an EEG depression (decreased amplitude, 2-3). Shortly after this period a second spike-train concomitant to the development of a generalized tonic-clonic seizure with wild running and jumping becomes apparent which finally results in a tonic extension of the hindlimbs (4) and death. The remaining tiny signal following brain death represents an ECG (R-spike) contamination. (b) After i.p. administration of bicuculline methobromide (BMB, 10 mg/kg) mice show trains of characteristic spikes and spike waves. (c) Administration of baclofen (20 mg/kg) resulting in sporadic occurrence of spiking activity. (d) Intrahippocampal electroencephalographic (EEG) recordings following i.p. administration of KA (30 mg/kg). (I): deep CA1 recording from a C57Bl/6J mouse for 2 h immediately after KA administration. At 30 mg/kg KA contiguous hippocampal seizure activity is observed occasionally interrupted by postictal depression (arrows). Ictal discharges are characterized by spike and/or spike-wave activity (see insets) in the delta- and theta-wave range (4–8 Hz). (II–IV) At days 1, 3, and 5 after injection 1 h CA1 EEG recordings illustrate declining but still continuous ictal discharges related to neuronal excitotoxic degeneration (with permission from [96, 97]).

A common field of application in EEG radiotelemetry is epilepsy research. Epilepsy models include acute and chronic pharmacological models as well as genetic models of epilepsy. In the following some pharmacological seizure models are described in more detail. Acute models of nonconvulsive absence-like seizures include i.p. administration of R/S-baclofen (Sigma, Germany), for example, at 20 mg/kg and bicuculline methobromide (BMB, Sigma, Germany), for example, at 10 mg/kg. Both substances, baclofen, acting pharmacodynamically as a GABA(B) receptor agonist, as well as bicuculline, which exerts antagonistic effects on GABA(A) receptors, can provoke spikes and spike waves in the ECoG based on the induction and maintenance of hypersynchronisation processes within the thalamocortical-corticothalamic circuitry. Figures 6(b) and 6(c) illustrate a surface EEG following the i.p. administration of R/S-baclofen and BMB at dosages described above.

Systemic administration of 4-aminopyridine (4-AP), for example, at a dosage of 10 mg/kg i.p. or pentylenetetrazole (PTZ), in mice can provoke generalized tonic-clonic seizures. The animals exhibit a typical progressive sequence of increasing seizure activity. Shortly after the injection, mice are hypoactive, followed by a mild, partial myoclonus mainly affecting the face, for example, vibrissal twitching, the head, and/or forelimbs. This state can evolve into a generalised clonus characterised by loss of upright posture, whole body clonus involving all four limbs, jumping, wild running, and, finally, a tonic extension of the hindlimbs.  Figure 6(a) illustrates a characteristic recording following 4-AP administration (10 mg/kg). At early stages of seizure development (myoclonus of the head, face, and forelimbs) the EEG contains only marginal EMG contamination. Following sporadic spike activity (*∗*) the generalised clonus initiates with a characteristic deflection (1) and a subsequent episode of continuous spike activity. Although this period is characterised by massive muscle activity due to the whole body clonus, spike activity of the brain can clearly be determined and EMG contamination is surprisingly low, indicating that the implantation procedure is capable of exhibiting electroencephalographic signals selectively even under generalised seizure conditions, when EEG signals might be expected to be masked by EMG artefacts. The first generalised clonus is followed by a typical short postictal depression (2-3) which is followed by a second generalized clonus which ended in a tonic extension of hindlimbs (4). Clearly, dose-effectiveness studies should be performed to identify the optimum dosage for a specific scientific question. In particular, optimum dosages can severely differ between genders, strains, and transgenic mouse lines. Control recordings should be performed prior to injection and control groups that have been administered a drug-free vehicle also have to be included.

Hippocampal seizure activity can be induced by kainic acid (KA) or N-methyl-D-aspartate (NMDA, Sigma). Both drugs can easily be dissolved in physiological 0.9% NaCl before injection. The non-NMDA receptor agonist KA is generally administered intraperitoneally at a dose of 10–30 mg/kg. As outlined above, pentylenetetrazole and 4-AP are typically used to provoke convulsive generalized tonic-clonic seizures. R/S-baclofen and *γ*-hydroxybutyrolactone are normally used to induce nonconvulsive absence like activity in mice. Hippocampal or complex partial seizures represent a third important seizure subgroup that can be acutely induced by various glutamate receptor agonists. Administration of KA, for example, causes a well-characterized hippocampal seizure syndrome (Figure 6(d)) that can be analyzed according to a slightly modified seizure score from [98]:* stage 1*, no behavioral change;* stage 2*, facial clonus;* stage 3*, forelimb clonus;* stage 4*, rearing;* stage 5*, falling;* stage 6*, status epilepticus;* stage 7a*, jumping, tonic seizure >30 s;* stage 7b*, jumping, tonic seizure 30–60 s;* stage 8*, maximum generalized seizure activity, respiratory arrest, and death. It should be noted that various seizure scores have been described in the literature that slightly differ from each other. Besides KA, hippocampal seizures can also be induced by i.p. administration of NMDA at a dose of 150 mg/kg. In NMDA treated mice, seizures developed through a sequence of paroxysmal scratching, hypermotility and circling, tonic-clonic convulsions, and, occasionally, death. The following semiquantitative scale can be used for the examination of seizure severity slightly modified according to [99]:* stage 0*, no response;* stage 1*, excessive grooming and paroxysmal scratching;* stage 2*, mild hypermotility;* stage 3*, extensive hypermotility and circling;* stage 4*, forepaw clonus and tail hypertonus;* stage 5*, generalized tonic-clonic convulsions;* stage 6*, status epilepticus;* stage 7*, death.

### 3.3. Seizure Activity following Chronic Administration of Proconvulsive/Psychoenergetic Drugs

**Figure 7:**
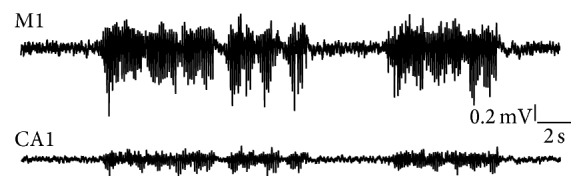
Radiotelemetric EEG recording in a rat model of mesial temporal lobe epilepsy. Limbic seizures are pharmacologically induced via a pilocarpine injection regime. This figure illustrates synchronous recording from the primary motor cortex (M1) as well as the hippocampal CA1 region from a rat at the age of 3 months. Ascending and descending spike/poly-spike trains are present in both deflections.

**Figure 8:**
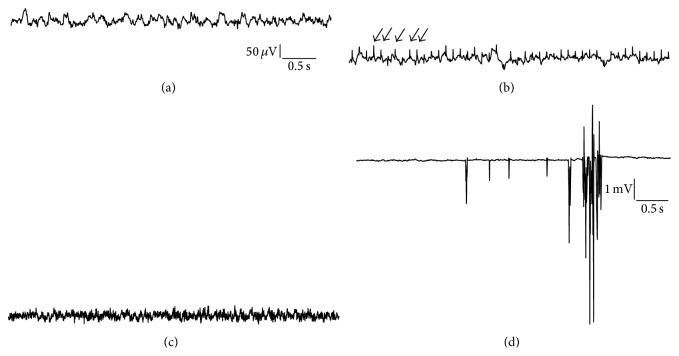
Electrocardiographic/electromyographic and system artefacts contaminating the EEG (deep electrodes (a)–(c), surface electrodes (d), vertical bar: 50 *μ*V in (a)–(c), and 1 mV in (d)). (a) Intrahippocampal EEG recording from a control mouse. (b) Damaged silicone insulation of the sensing leads as well as ossification processes originating from the edge of drilled holes can result in dramatic contamination of electroencephalographic recordings. Note the regular pattern of interfering ECG signal, that is, R-spikes (arrows). Importantly, ECG contamination cannot be completely avoided but the implantation procedure presented here will reduce it to a minimum. (c) Electromyographic contamination of the EEG characterized by high frequency activity. (d) Artefacts can also originate from cross-talk between receiver plates or from electrical noise evolving from room lights or various other electrical devices that are close to the receiver plates. An effective way of preventing the system picking up noise is to shield receiver plate and home cage using a ventilated cabinet or a Faraday cage (with permission from [85]).

One of the most popular chronic seizure models is the pilocarpine model of mesial temporal lobe epilepsy (mTLE) in rats. Various pilocarpine administration regimes have been described in the literature that lead to a more or less stable and life-long hippocampal seizure phenotype.  Figure 7 illustrates examples of cortical and hippocampal EEGs in the rat pilocarpine model (see also supplementary video in Supplementary Material available online at http://dx.doi.org/10.1155/2016/8213878). It should be noted that EEG artefacts can sometimes mimic ictiform discharges (Figure 8). Thus, special attention has to be paid to reduce ECG, EMG, and externally induced EEG signal disturbance.

### 3.4. Seizure Activity in Genetically Engineered Mouse Models

Animal models provide a means to investigate the fundamental mechanisms of abnormal electrical discharge that occur during seizure activity. Understanding the pathogenesis of seizures and epilepsy, therapy development has greatly benefited from these models. However, it should be noted that reductionist approaches also exist, making use of models in* Drosophila melanogaster*,* Caenorhabditis elegans*, and zebrafish Danio rerio. Studies of seizures/epilepsy in mutant mice provide a framework for understanding the critical features of the brain that regulate excitability and hyperexcitability. These, and as yet undiscovered, mouse mutants will continue to serve as the foundation for basic epilepsy research when displaying the principles of homology, isomorphism, and predictability regarding human diseases. A considerable level of effort is still devoted to the discussion of animal models of epilepsy and consensus on which models best reflect the human condition is difficult to achieve. Clearly, emerging models are pushing our perspective towards novel genetic, cellular, and anatomical hypotheses for the etiopathogenesis of epilepsies.

About 100 genetically modified mouse models were reported to exhibit an epileptic phenotype in the current literature. In the past decade, the number of genetically modified mice exhibiting seizures or reduced sensitivity to convulsant manipulations has risen exponentially, with no clear endpoint in sight. To a large extend, these mutant mice feature a general inactivation or “knockout” of a specific gene and confirm much of what has already been known about the sophisticated excitation-inhibition balance in the CNS. New genetically engineered mice provide additional insights into cellular mechanisms underlying seizure generation, genetic interactions that exacerbate seizure phenotype, and neurodevelopmental influences. From these mice candidate genes of ion channels, postsynaptic receptors, and genes required for neuronal migration were identified. EEG radiotelemetry has been one of the most effective techniques in the* in vivo* characterization of these mouse models as it does not only allow for screening for spontaneous seizure activity but also following pharmacological provocation. In addition, EEG radiotelemetry enables the recording under special experimental, that is, behavioral settings under which seizures might occur and thus proofs to be a most valuable tool in the field.

## 4. Analysis of Telemetric EEG Recordings

Analytical approaches in EEG radiotelemetry are a sophisticated field and are beyond the scope of this presentation. Similar to self-made transmitter systems many scientists have written their own home-made programs to analyze EEG data. It is also possible to analyze data collected with self-made recording systems using well-established analysis software depending on the data format. Most commercially available telemetry systems offer specific modules for frequency methods like fast Fourier transformation (FFT) analysis, automated seizure detection, or automated sleep analysis. Telemetry enables researches to acquire huge amounts of EEG data and automated analysis systems provide an easy and elegant way to analyze these data sets efficiently. However, special attention has to be paid to the configuration settings of automated analysis systems and it seems recommendable to compare software based analysis with visual/manual analyses of EEG data. Proper blinding is mandatory in both cases.

Although tools for FFT-based EEG analysis are generally provided, special scientific questions might require the development of new analytical tools. An example is given below. It illustrates the establishment of a novel analytical tool to detect highly synchronous hippocampal theta oscillations using deep intrahippocampal CA1 recordings following administration of urethane [93]. Data segments with a length of 30 min each are extracted from the preinjection phase and the posturethane phase. Data segments are analyzed using complex Morlet wavelets to calculate both frequency and amplitude of oscillations. The complex Morlet wavelet is defined by Ψ(*x*) = (*πb*)(−1/2)exp⁡(2*iπcx*)exp⁡(−*x* ^2^/*b*), where *b* is the bandwidth parameter, *c* the center frequency, and *i* the imaginary unit. This wavelet or similar ones have often been applied in the literature to study EEG data, as they guarantee optimal resolution in both frequency and time [100]. In our case, the bandwidth parameter and center frequency are both set to 3 in order to particularly weight the frequency resolution to distinguish frequency differences on the 0.1 Hz level, but not to neglect a sufficient time resolution. EEG data are analyzed in the frequency range of 0.2–12 Hz with a step size of 0.1 Hz, thus including the typical delta, theta, and alpha frequency ranges. In order to apply the wavelet technique for extraction of theta-oscillatory segments, we developed a task-adjusted detection criterion [93, 94]. This theta detection method imitates the standard visual inspection of theta oscillations and is mainly based on a complex elaboration of the frequency architecture of theta activity. The theta oscillation detection criterion is equal to the quotient of the maximum amplitude in the theta frequency range (3.5–8.5 Hz) and the maximum amplitude in the upper delta frequency range (2–3.4 Hz) for a time window of 2.5 s. With a ratio above 1.5, that is, the maximum theta amplitude is at least 50% higher than the amplitude in the upper delta band, the related 2.5 s EEG segment is classified as a theta oscillatory epoch. An interval of 2.5 s represents a minimal duration for a theta oscillation and prevents from false negative detections of certain noisy epochs and lies within the range definitions of [101]. The upper delta frequency range serves as a control frequency band because physiological relevant delta activity appears during nontheta epochs, for example, during slow-wave sleep, which is highly damped during theta activity.

## 5. Concluding Remarks

EEG radiotelemetry has turned out to be a most valuable tool in basic research and drug research and development, particularly in neuropsychiatric, neurodegenerative, and epilepsy research. In addition, it significantly contributes to the 3R strategy. EEG radiotelemetry is per se a most sensitive tool harboring tremendous analytical potentials. As an example, complex analysis of EEG time-frequency patterns might result in predictive biomarkers in neurodegenerative diseases in the future. However, it is always mandatory to pay specific attention to potential pitfalls of this sophisticated technique and to factors that might influence radiotelemetric results and which might cause sporadic or systematic bias in telemetry data.

## Supplementary Material

The supplementary video illustrates a simultaneous video-EEG recording from a pilocarpine treated rat. A pilocarpin injection regime had been applied to induce medial temporal lobe epilepsy.

## Abbreviations

CNS: Central nervous system
EEG: Electroencephalogram
ECoG: Electrocorticogram
EMG: Electromyogram
ECG: Electrocardiogram
i.p.: Intraperitoneal
KA: Kainic acid
NMDA: N-Methyl-D-aspartate
s.c.: Subcutaneous.
